# Progressive Assessment of Ischemic Injury to White Matter Using Diffusion Tensor Imaging: A Preliminary Study of a Macaque Model of Stroke

**DOI:** 10.2174/1874440001812010030

**Published:** 2018-03-30

**Authors:** Xiaodong Zhang, Yumei Yan, Frank Tong, Chun-Xia Li, Benjamin Jones, Silun Wang, Yuguang Meng, E. Chris Muly, Doty Kempf, Leonard Howell

**Affiliations:** 1 Yerkes National Primate Research Center, Emory University, Atlanta, Georgia 30329; 2 Department of Radiology, School of Medicine, Emory University, Atlanta, Georgia 30322; 3 Department of Psychiatry and Behavioral Sciences, School of Medicine, Emory University, Atlanta, Georgia 30322

**Keywords:** Stroke, Nonhuman primate, DTI, MCA occlusion, Infarct evolution, Ischemic injury

## Abstract

**Background:**

Previous Diffusion Tensor Imaging (DTI) studies have demonstrated the temporal evolution of stroke injury in grey matter and white matter can be characterized by DTI indices. However, it still remains not fully understood how the DTI indices of white matter are altered progressively during the hyperacute (first 6 hours) and acute stage of stroke (≤ 1 week). In the present study, DTI was employed to characterize the temporal evolution of infarction and white matter injury after stroke insult using a macaque model with permanent ischemic occlusion.

**Methods and materials:**

Permanent middle cerebral artery (MCA) occlusion was induced in rhesus monkeys (n=4, 10-21 years old). The brain lesion was examined longitudinally with DTI during the hyperacute phase (2-6 hours, n=4), 48 hours (n=4) and 96 hours (n=3) post-occlusion.

**Results:**

Cortical infarction was seen in all animals. The Mean Diffusivity (MD) in lesion regions decreased substantially at the first time point (2 hours post stroke) (35%, p <0.05, compared to the contralateral side) and became pseudo-normalized at 96 hours. In contrast, evident FA reduction was seen at 48 hours (39%, p <0.10) post-stroke. MD reduction in white matter bundles of the lesion area was much less than that in the grey matter during the hyper-acute phase but significant change was observed 4 hours (4.2%, p < 0.05) post stroke . Also, MD pseudonormalisation was seen at 96 hours post stroke. There was a significant correlation between the temporal changes of MD in white matter bundles and those in whole lesion areas during the entire study period. Meanwhile, no obvious fractional anisotropy (FA) changes were seen during the hyper-acute phase in either the entire infarct region or white matter bundles. Significant FA alteration was observed in entire lesion areas and injured white matter bundles 48 and 96 hours post stroke. The stroke lesion in grey matter and white matter was validated by pathological findings.

**Conclusion:**

The temporal evolution of ischemic injury to the grey matter and white matter from 2 to 96 hours after stroke onset was characterized using a macaque model and DTI. Progressive MD changes in white matter bundles are seen from hyperacute phase to acute phase after permanent MCA occlusion and temporally correlated with the MD changes in entire infarction regions. MD reduction in white matter bundles is mild in comparison with that in the grey matter but significant and progressive, indicating it may be useful to detect early white matter degeneration after stroke.

## 1 INTRODUCTION

Conventional MRI and Diffusion-Weighted Imaging (DWI) can provide valuable information for early detection of ischemic brain damage and to identify stroke lesion volume and territory [1-3]. Diffusion tensor imaging (DTI) allows for the non-invasive measurement of *in vivo* 3D diffusion of water molecules in brain tissues and has been demonstrated to be a promising noninvasive method to access the white matter integrity [4, 5]. Quantitative analysis of DTI indices has shown promising to evaluate pathological changes in ischemic tissues including grey matter and white matter in which DTI indices show a different pattern of evolution in animal models and patients [6-8]. The white matter integrity changes during acute (≤ 1 week) and chronic (> months) stages of stroke disease have been investigated previously in stroke patients [9-17], indicating white matter integrity alterations are closely associated with the functional impairment and recovery after stroke. Meanwhile, the discrepancy between the infarct volume and functional outcome in stroke patients is suggested partly due to the white matter degeneration after stroke onset [18, 19].

Most MR imaging studies focus on grey matter injury of acute stroke, and the temporal evolution of DTI indices are well studied and the findings are generally consistent. A few DTI studies have been performed to characterize white matter injury during hyperacute and acute stroke in rats with MCA occlusion [8, 20], stroke patients [21], and r-tPA treated patients [22]. The temporal changes of fractional anisotropy (FA) and diffusivity indices in cortex and ipsilesional corpus callosum in rats are well demonstrated from 2 hours to 6 or 8 weeks after transient MCA occlusion. Interestingly, FA recovery was seen in rats but not in patients during the subacute phase (>1 week) of stroke.

Rodent models of stroke offer tremendous information in understanding the ischemic effects on neuron function and survival and developing thrombolytic, neuroprotective, and restorative therapies [23]. However, rodents are essentially different from human regarding brain anatomy, cerebral metabolism, behavior and life span. In particular, rodents have little white matter. In contrast, the Nonhuman Primate (NHP) brain is gyrencephalic, structurally and functionally similar to the human brain, and can provide an unparalleled platform for drug testing and mechanistic studies of stroke disease than currently available rodent models [24-26]. The NHP models are recommended for preclinical neuroprotection studies of stroke by the Stroke Therapy Academic Industry Roundtable (STAIR) committee [25]. Therefore, it is clinically translational to use a non-human primate model to evaluate white matter injury during the acute stroke and examine its relationship with the infarction evolution. We hypothesized the diffusivity property of white matter bundles was altered following stroke insult. In the present study, DTI was applied to characterize infarction and white matter injury longitudinally using a macaque model of ischemic stroke.

## 2 MATERIALS AND METHODS

Adult female rhesus monkeys (n=4, 10-21 years old, 6.9-9.3 kg) were utilized in the present study. The demographic data and clinical histories of the animals were listed in Table (**1**). Permanent Middle Cerebral Artery (MCA) occlusion was induced using an interventional approach [3, 27]. The entire procedure was reported previously [28, 29]. Briefly, the animals were maintained under 1-2% isoflurane anesthesia throughout stroke surgery, and placed on a temperature-controlled heating pad, positioned, and restrained in the “supine” position over the entire procedures. Animal physiology parameters (such as heart rate, respiratory rate, isoflurane level, rectal temperature, blood pressure, end-tidal CO_2_, O_2_ saturation) were monitored continuously and maintained [30]. The occlusion was conducted with several separate pieces of 4-0 or 3-0 silk suture, measuring in various lengths (3-25 mm) to cause occlusion of angiographically selected MCA branches. Stroke occlusion was confirmed angiographically during surgery. The animals were moved into the MRI scanner within 30 minutes after MCA occlusion and scanned for 6-7 hours. The animals were rescanned at 48 hours (n = 4) and 96 hours (n=3) post-stroke.

Stroke animals were under intense care by trained veterinary staff in our facility in the first 24 hours to ensure each animal was in proper condition and receiving immediate medical care if needed. After the first 24 hours, stroke animals were examined a minimum of twice a day until they were sacrificed. A 24-hour surveillance video camera was also used to continuously monitor the stroke animals until their last MRI scans. The surviving animals were euthanized immediately after the 48-hour (n=1) and 96-hour (n=3) MRI scan. The details about the animal care in the present study were reported previously [28].

All procedures were approved by the Institutional Animal Use and Care Committee (IACUC) at Emory University in a facility fully accredited by Association for Assessment and Accreditation of Laboratory Animal Care (AAALAC) and in compliance with the Animal Welfare Act and the Public Health Service Policy on Humane Care and Use of Laboratory Animals.

### 2.1 MRI Examination

After the ischemic occlusion surgery, animals were moved immediately into a 3T MRI clinical scanner (MAGNETOM TIM Trio, Siemens Healthcare, Erlangen, Germany) and scanned with a phased-array 8-channel knee coil (*In vivo Inc.*, FL) for 6-7 hours, then re-scanned at 48 and 96 hours post occlusion. DTI data was acquired with a single-shot EPI sequence with the parameters: TR = 5000 ms / TE = 80 ms, b-value = 1000 s/mm^2^, 30 gradient directions, 1.5 mm isotropic resolution, 4 repetitions. Also, MR angiography (MRA), T1-weighted images, T2-weighted images were acquired for stroke lesion validation purpose [28]. In addition, each animal received a pre-scan performed one week before surgery for screening purpose. When scanning an animal, the anterior commissure – posterior commissure line (AC-PC line) was used as a reference for defining the central slice location and orientation in every scan. Animals were sacrificed immediately after their last scans for histology.

### 2.2 Data Processing

DTI data were prepared with the FSL software (University of Oxford) for eddy-current distortion correction, co-registration, and then processed with the DTI-Studio software (Johns Hopkins University) for calculating mean diffusivity (MD) and fractional anisotropy (FA). For each monkey, the stroke-injured regions were identified with DWI images and MD maps and cross-validated with corresponding T2-weighted images. Stroke lesion regions during hyper the hyperacute phase were derived from DWI images using the threshold (mean + 2 × standard deviation (SD)) of the DWI intensity on the contralateral side. Lesions at 48 and 96 hours were from T2W images in comparison to the contralateral side using manual tracing. FA maps, DWI and T2-weighted images were used as a reference to structurally define the ROIs for white matter bundles at each time point. The white matter bundles seen at the first time point on FA map and within the infarct territory at last time point of each animal were selected as regions of interest (ROIs). The contralateral side of the brain of the same animal was used as a reference for comparison purpose. MD and FA values in the entire lesion area and the fiber bundle ROIs were calculated. The MD and FA in the lesion side and the corresponding contralateral side were compared with paired *t* test at each time point.

Pearson correlation analysis was performed between the averaged FA or MD values in the entire infarction regions of all animals at each time point and those in the white matter fiber tracts using SPSS 22.0.

### 2.3 Histology

All animals were euthanized without recovery from anesthesia after their last MRI scans by pentobarbital overdose and immediately intracardially perfused with saline followed by 10% buffered formalin according to well-established protocols approved by the Emory IACUC. Brains were removed and immersed in 10% buffered formalin. The brains were then blocked and sectioned at 50 µm using a freezing microtome. Selected sections were then stained with Hematoxylin and Eosin (H&E) and luxol fast blue to identify the stroke lesion and white matter damages.

## 3 RESULTS

Stroke lesions were observed in the cortical regions of all animals. The MCA occlusion in each subject was confirmed with MR Angiography (data not shown) and T2-weighted images. The FA and MD maps, and DWI images of one stroke monkey (RFA5) at each time point are shown in Fig. (**1**). The temporal changes of FA and MD in whole lesion regions and injured white matter during the hyperacute phase (2-6 hours) and at 48 and 96 hours were further evaluated.

**Fig. (3):**
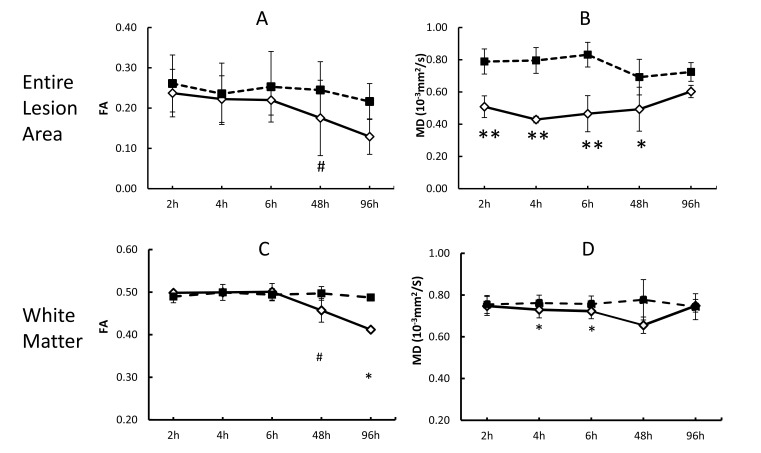
Demonstration of temporal changes of FA and MD in entire lesion regions and white matter bundles within the lesion areas. A) Progressive changes of FA, and B) MD in the entire lesion regions after stroke insult. C) Progressive changes of FA, and D) MD in the white matter bundles after stroke insult. h: hours post stroke; solid line: stroke side; dash line: contralateral side; *, p<0.05, **, p<0.01, paired student’s t test between stroke side and contralateral side.

The quantitative changes of FA and MD in injured white matter bundles of each animal are exhibited in Fig. (**2**), illustrating inter-subject differences of FA and MD during stroke evolution. Also, their averaged changes of FA and MD are exhibited together with those in the entire lesion regions (mostly grey matter) for comparison purpose Fig. (**3**). In the entire lesion regions, FA did not show obvious changes during the first 6 hours after stroke onset Fig. (**3A**). Evident FA reduction (39%, p< 0.1) was observed 48 hours post stroke. As expected, MD decreased substantially at the first time point (2 hours post stroke) (35%, p < 0.05) and during the remaining hyperacute phase and at 48 hours post occlusion and approximated to baseline values at 96 hours post stroke (Fig. **3B**).

The progressive changes of MD and FA in injured white matter bundles are shown in Figs. (**3C** and **3D**) . No evident FA changes were seen during the hyperacute phase of stroke. Evident FA reduction (8.0%, p < 0.1) was observed at 48 hours post stroke and FA kept decreasing at 96 hours post stroke Fig. (**3C**). Obviously, the MD values in the white matter bundles decreased significantly 4 hours post stroke (4.2%, p< 0.05), and recovered to the baseline (pseudo-normalization) at 96 hours post stroke Fig. (**3D**). No correlation was seen between the FA values in the entire lesion regions and those in affected white matter bundles during the entire study period (p=0.17, Fig. (**4A**). However, the MD values in the entire lesion regions were temporally correlated with those in injured white matter fibers significantly during the entire study period (p=0.03) Fig. (**4B**)and also in the hyper-acute phase (p < 0.01).

### 3.1 Stroke Volume Evolution

The mean infarct volume changed from 3.1±1.9ml at 6 hours post stroke (by DWI, n=4) to 4.7±2.8ml at 48 hours (n=4) and 4.7±2.3ml at 96 hours (n=3) by T2-weighted images. No obvious lesion volume increase was observed from 48 to 96 hours post stroke.

### 3.2 Histology

**Fig. (5):**
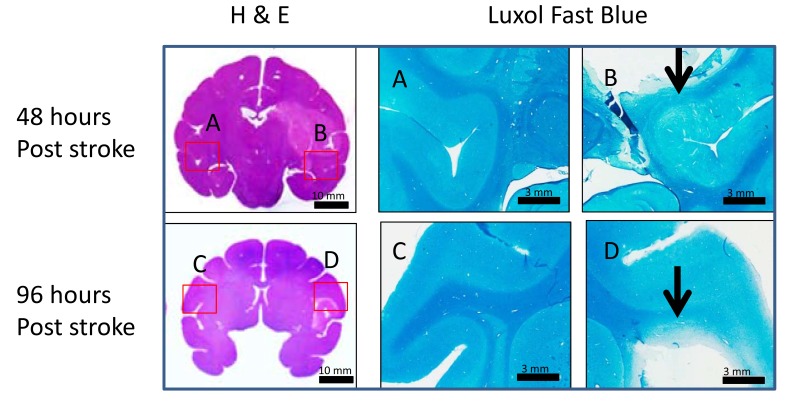
Illustration of H & E and Luxol fast blue stained images. H & E (left column) and Luxol fast blue staining images (right column) in monkey brains 48 (top row) and 96 hours (bottom row) after MCA occlusion. Scale bars are shown at the right bottom of each picture. The corresponding regions of luxol fast blue staining pictures are labeled in the H&E staining whole brain pictures with A, B, C, and D (left: contralateral, right: ipsilateral) . Arrows point to abnormal white matter fibers.

Stroke lesions from two stroke brain samples (RRI3 and RJJ3) obtained at 48 hours and 96 hours post occlusion are demonstrated respectively on the H&E staining slices. White matter fiber injury was seen at 48 and 96 hours post occlusion using luxol fast blue myelin staining (marked with arrows) (Fig. **5**) .

## 4 DISCUSSION

The present study examined the temporal evolution of fractional anisotropy and mean diffusivity in lesion regions and injured white matter fiber tracts from 2 to 96 hours after stroke insult. No obvious FA changes were seen in either the entire infarct regions or white matter fibers during the hyperacute phase as expected. In contrast, significant MD reduction was seen immediately post stroke in the entire infarct region and at 4 hours in white matter fibers. White matter bundles were impaired dramatically 48 hours post stroke as indicated by substantial FA reduction. Also, the present study demonstrates that progressive MD changes in stroke lesion regions and white matter bundles are correlated temporally during the hyperacute phase and entire study period, indicating the temporal relationship between the infarct evolution and white matter degeneration in the pathophysiological cascade of events from the initial ischemic insult to acute stage of stroke.

Stroke results in regional brain tissue damage and grey matter is usually the focus in most preclinical and clinic studies. Evident MD reduction is generally seen in the infarct area immediately after stroke onset. The evolution pattern in diffusivity changes has been used to characterize the development of acute stroke injury [31, 32]. Such MD decrease after stroke insult is thought to be due to cytotoxic edema that results from cellular energy failure and the subsequent shift of water into the intracellular compartment where diffusion is more restricted [32]. In contrast, FA is not recognized as a sensitive parameter to characterize the brain tissue injury during the early phase of stroke. Previous studies in rats reported increased FA in the first hour of ischemia and then declined during the acute phase [33]. FA elevation was also seen in the deep grey matter and white matter of patients with hyperacute stroke [22]. However, no evident or significant FA changes during the hyperacute phase of stroke were reported in previous studies using macaques with permanent and transient MCA occlusion [6] and marmosets [19], in agreement with our present results.

MD reduction was previously observed in acute white matter injury [34]. The temporal evolution of the DTI indices in different brain regions including cortex, subcortex, and corpus callosum has been investigated systematically in rats with transient MCA occlusion [8], in which MD reduction in corpus callosum was significant during hyper acute phase (2-3.5 hours) and obvious MD increase was seen one and two days post stroke. As reported in the present study, the progressive changes of MD in white matter bundles of macaques decreased gradually after stroke insult, and evident MD recovery was seen at 96 hours post stroke. Also, significant MD reduction was observed 4 hours, but the magnitude of MD changes is much smaller than that in the entire infarct regions (mainly grey matter) (4.2% vs 46.2% at 4 hours post stroke). Obviously, the MD reduction in white matter was gradual and couple hours lagged behind the MD decreasing in grey matter, suggesting the resistance to ischemia is higher in white matter than that in the grey matter [35]. In addition, the MD changes of white matter in stroke macaques are in agreement with Tamura et al’s report in stroke patients [21].

Pitkonen et al’s report showed FA recovery in corpus callosum of rats one week after temporary ischemic stroke [8]. In contrast, such FA recovery was not observed in our study of macaques from hyper acute phase to 96 hours post-stroke. Such discrepancy of FA evolution pattern in white matter most likely is caused by the difference between the reperfusion and permanent model of stroke. In addition, our present DTI results in macaque brains showed that temporal MD changes in the entire infarct region (grey matter) correlated with that in white matter fibers during the hyper-acute phase (R^2^ = 0.40, P < 0.01) or entire study period (R^2^=0.51, P = 0.03), indicating the temporal relationship between the infarct evolution and white matter degeneration in the pathophysiological cascade of events from hyperacute phase to acute phase after stroke insult.

In addition, as shown in Fig. (**2**), MD reduction was observed consistently in injured white matter fibers of each animal during the hyper-acute phase while the FA evolution pattern varied changed from animal to animal. The results further suggest MD in white matter is a robust biomarker to characterize the fiber degeneration following stroke insult in hyperacute phase. The MD reduction was also seen at 48 h post stroke as expected. However, the MD recovery of each animal was not consistent at 96 h post stroke, suggesting the timing of pseudo-normalization is different for each case.

In the current study, the animals were maintained under isoflurane anesthesia for about 7 hours during the surgical day. As isoflurane can modulate blood flow in the brain [30, 36-38], the stroke lesion may be affected by the isoflurane anesthesia. Prior rodent studies suggest that isoflurane preconditioning and post-treatment may have the neuroprotective effects in stroke brains [39, 40]. But the protective effect is conditional as shown in an awake mice study of stroke [41], and the neuroprotective effect was not seen in prior primate study of stroke [42]. Therefore, more studies are warranted to examine the neuroprotective effects of isoflurane in stroke.

To date, the only FDA-approved post-stroke intervention is intravenous thrombolysis using recombinant tissue plasminogen activator (rt-PA) within ~4.5 hours after the symptom onset [43, 44]. However, such thrombolysis strategy is very limited in clinical practice due to the short treatment time window and increased hemorrhage complication [45, 46]. As a result, the thrombolysis strategy is restricted to only a small proportion of patients.

Neuroprotective therapies have been explored extensively and demonstrated neuroprotection of stroke lesion in prior preclinic studies. Even though all are failed in clinical trials, they are still being explored [47, 48] and remain to be a promising strategy of acute stroke therapy [49, 50]. In comparison to rodent models which have little white matter, NHPs mimic most aspects of human, allowing for testing the effects of neuroprotective agents in higher-order gyrencephalic brains [24]. Due to the role of white matter bundles in brain structural connectivity and axonal remodeling in stroke recovery [51] and cell treatment [52, 53], the current findings of the evolution pattern of white matter bundles following stroke insult may be used to characterize the treatment effects of neuroprotective agents on white matter during acute stroke.

## CONCLUSION

The temporal evolution of fractional anisotropy and mean diffusivity in ischemic tissues and white matter bundles after stroke insult were revealed using a macaque model with permanent MCA occlusion. Significant MD alteration in infarct areas and injured white matter bundles was observed during the hyperacute phase of stroke. The mild MD changes in injured white matter bundles are temporally correlated with those in whole lesion regions (grey matter) during the hyper-acute phase and the entire study period (up to 96 hours post stroke). The results suggest MD of white matter bundles may be useful to detect early white matter degeneration in acute stroke.

## Figures and Tables

**Fig. (1):**
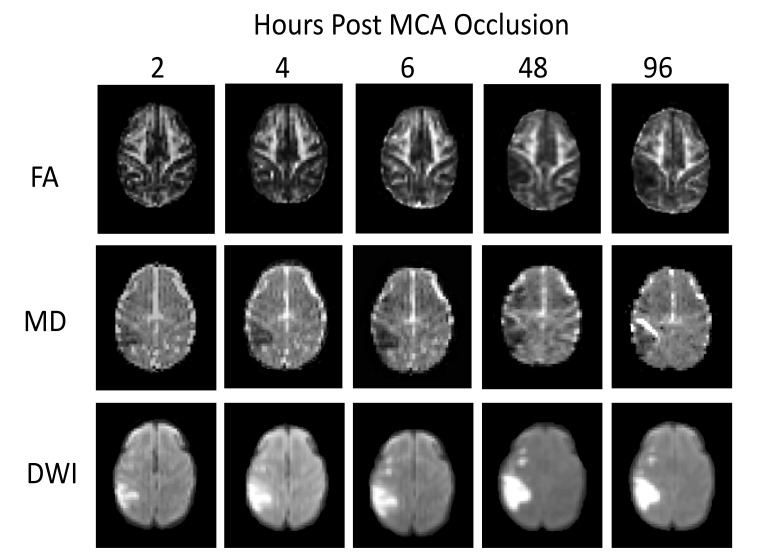
Demonstration of temporal evolution of fractional anisotropy (FA), mean diffusivity (MD), and infarction (DWI) after stroke insult in a macaque brain (RFA5).

**Fig. (2):**
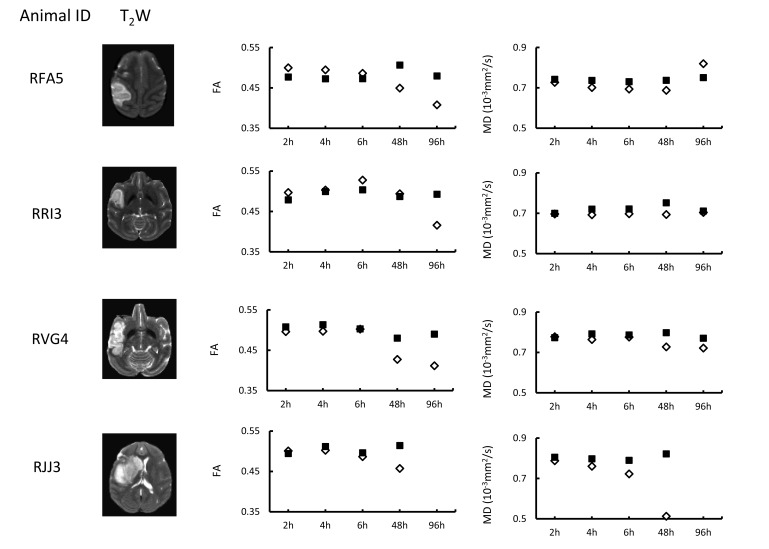
Demonstration of temporal changes of fractional anisotropy (FA) and mean diffusivity (MD) in injured white matter bundles after stroke insult in each animal. The stroke infarct in each animal was illustrated in the corresponding T2-weighted images. Square: contralateral side; Diamond: ipsilateral side. h: hours post stroke.

**Fig. (4):**
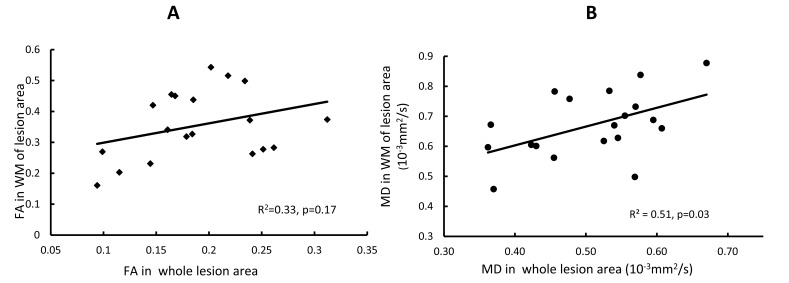
Relationship between the MD and FA in the entire lesion region and those in injured white matter fibers (WM). A) correlation between FA in the entire lesion region and those in white matter (WM) fibers at all time points. B) correlation between MD in the entire lesion region and those in WM fibers at all time points.

**Table 1:** Demographic data of stroke monkeys.

**Monkey ID**	**Sex**	**Age (years)**	**Body Weight (kg)**	**Clinical History**
RJJ3	Female	21.5	8.2	Stem cell study. Stem cells were infused into the animal’s fetus.
RFA5	Female	18	9.3	Vaccine study for control. Surrogate for pregnancy.
RRI3	Female	21.5	8.2	From breeding colony. Used on an aging study. No compromising intervention.
RVG4	Female	10.5	6.9	Malaria infected. No infection at time of stroke. Also used on a vaccine study. Surrogate for pregnancy.

## LIST OF ABBREVIATIONS

MRI: = Magnetic Resonance Imaging
EPI: = Echo-planar Imaging
DTI: = Diffusion Tensor Imaging
DWI: = Diffusion Weighted Imaging
MD: = Mean Diffusivity
FA: = Fractional Anisotropy
DSI: = Diffusion Spectrum Imaging
MCA: = Middle Cerebral Artery
NHP: = Non-human Primate
IACUC: = Institutional Animal Care and Use Committee
AAALAC: = Association for Assessment and Accreditation of Laboratory Animal Care
MRA: = MR angiography
TE: = Echo Time
TR: = Repetition Time
FOV: = Field of View
ROI: = Region of Interest
H&E: = hematoxylin and eosin
